# Characterization of the goat feeding system among rural small holder farmers in the semi-arid regions of Uganda

**DOI:** 10.1186/s40064-015-0961-3

**Published:** 2015-04-18

**Authors:** Dorothy Kalule Nampanzira, John David Kabasa, Sara Agnes Nalule, Immaculate Nakalembe, John Robert Stephen Tabuti

**Affiliations:** Department of Livestock and Industrial Resources, Makerere University, P. O. Box 7062, Kampala, Uganda; Department of Biosecurity, Ecosystems & Veterinary Public Health, Makerere University, P. O. Box 7062, Kampala, Uganda; Department of Environmental management, Makerere University, P. O. Box 7062, Kampala, Uganda; Department of Wildlife and Aquatic animal resources, Makerere University, P. O. Box 7062, Kampala, Uganda; Department of Bio molecular resources and Biolab Sciences, Makerere University, P. O. Box 7062, Kampala, Uganda

**Keywords:** Tethering, Goat feed resources, Feeding calendar, Important value index

## Abstract

Goats (*Capra hircus*) are widely distributed in Africa and Asia, and are important to the subsistence and economic livelihoods of many people in these areas. The goat feeding system among rural small holder farmers in Buyende district (Uganda) was characterised by determining the goat rearing practices, feed resources fed on by goats and availability of browse species mentioned by small holder farmers. Data was gathered using ethnobotanical and ecological approaches. Results from the ethnobotanical survey revealed that farmers were rearing indigenous goat breeds that are managed by tethering in natural pastures during the rainy season but free ranging during the dry season (i.e. when no crops are susceptible of damage). Major challenges facing goat production in the study area were diseases, shortage of land and inadequate pastures. The reduction of grazing land due to crop farming, has led to tethering of animals which in turn leads to restricted feeding. Goats were known to feed on 48 plant species distributed in 18 families and 39 genera dominated by trees and shrubs*.* Browse species were known to stay longer in the dry season when the grass and herbaceous species were no longer available. The most frequently mentioned browse species were *Ficus natalensis*, *Harrisonia abyssinica*, *Acalypha psilostachya*, *Artocarpus heterophyllus* and *Lantana camara* while *Panicum maximum* and *Impeata cylindrica* were the most mentioned herbaceous species. 31 browse species were encountered in the ecological survey. These were dominated by *Combretum molle*, *L. camara*, *A. zygia*, *M. indica*, and *Albizia coriaria.* In conclusion, the rearing practices of goats in Buyende district are comprised of indigenous goats tethered in natural pastures especially browses which stay longer through the dry season. However, most of the preferred browses are rare according to the computed IVI (i.e. less than 30%).

## Introduction

Nutrition plays an essential role in goat farming systems. In developing countries, these systems are characterised by low input of poor quality pastures that contribute to inadequate feeding and nutrition (Ben Salem and Smith 2008), and productivity is low (Thomas and Rangnekar 2004). This is in turn aggravated by the rearing practice that is mainly characterized by tethering of indigenous breeds in natural pastures (Rumosa Gwaze *et al*. 2009). Tethering is common in areas where land and forage production are limiting factors (Kosgey 2004). Improving feeding and nutrition, and maximising the use of the available feed resources should be the main target considered when enhancing goat productivity.

Pastures in semi-arid countries are subject to seasonal variability, with the rain season affecting availability and quality of forage (Fadel Elseed *et al*. 2002). In the dry season, goats rely on browse species for their nutrition (Kabasa *et al*. 2004). The nutritive value of most browse species is known to be high, except for the presence of secondary metabolites, but with low variation over time compared to grasses (Fadel Elseed *et al*. 2002). Crop residues and food by-products are also present during the dry season. However, these feed resources are low in crude protein, high in fibre and less digestible. Availability of feed resources and rearing practices are important aspects when planning for sustainable livestock feeding systems in a community. The objective of this study was to determine the goat rearing practices, preferred goat feed resources and their availability in Buyende District, Uganda.

## Materials and methods

### Study area

This study was conducted in Buyende District in Uganda located between 1°14′-1°29′ N and 33°16′-33°28′ E. This study was conducted in two of the five sub-counties of Buyende district that are Kidera and Nkondo. The district lies at an average altitude of 1,083 m above sea level in a relatively flat landscape. The area has a bimodal rainfall pattern with March to May and September to November as the first and second rain seasons respectively. The average annual rainfall is 1200 mm. The mean daily maximum and minimum temperatures of the area are 28°C and 16°C, respectively. The soils of the district are comprised of grey to yellowish or reddish brown sandy loams, and clays (Ollier and Harrop 1959). The original vegetation of Buyende District consisted of dry combretum savannas, with *Combretum-Terminalia-Loudetia*, *Combretum-Hyparrhenia* and *Combretum-Acacia-Hyparrhenia* savanna plant communities (Langdale-Brown *et al.*^1964^). Most of the original vegetation has been replaced with crops and the current land use consists almost entirely of small scale farmland. The district has a human population of about 270,000. The main livelihood of the people in this area is crop and livestock farming. Fishing is practiced to a small extent. The major types of crops grown are sweet potatoes, maize and cassava, and the type of livestock raised comprises chicken, goats and cattle (UBOS 2013).

### Data collection

Data for this project was collected using ethnobotanical and ecological approaches. At the onset of the study discussions were held with the local area politicians and technical staff including the District Veterinary Officer and the sub-county Veterinary Officer. During these discussions the study objectives were explained and discussed. From these discussions, Kidera and Nkondo Sub-counties were recommended for the study because they had the most cattle and goats in the district. We also requested for and received permission to access the study area. Local leaders were requested to sensitize farmers about the study and to obtain farmers’ permission to access their land during the surveys. In all ecological surveys we were accompanied by a local leader. A preliminary study was then conducted to test the tools to be used in data collection.

### Ethnobotanical survey

Household respondents were chosen using the snowballing method. In every parish one goat farmer was identified with the help of the sub-county Veterinary Officer. The selected farmer in turn identified the next farmer to visit. In this way, 139 respondents (72 males and 67 females) participated in the survey. The respondents were distributed within 14 villages. Data was collected using a mixed open and closed-ended questionnaire in face-to-face interviews. The interviews were conducted in Lusoga, the dominant local language spoken in the study area. The questionnaire covered the following broad themes: goat rearing practices, feed resources eaten by the goats, techniques used in improving feed resources and seasonal availability of the feed resources.

We also conducted four focus group discussions (FGDs), each with seven participants. The aim of the FGDs was to; prioritize browse species that farmers consider most important and most browsed by goats; construct a seasonal availability calendar of the feed resources and verify data from the questionnaire interviews. Lastly, we used the technique of direct observations using a checklist to verify the goat rearing practices in the area. Plant voucher specimens of all species mentioned in the study were collected and taken to Makerere University Herbarium (MHU), for identification and are deposited there. Species nomenclature follows the Flora for Tropical East Africa. Species names were checked for accuracy using the TROPICOS database (http://www.tropicos.org/). Data was summarized into percentages, and presented in tables and graphs.

### Ecological survey

The ecological survey was conducted to determine the availability of species mentioned in the ethnobotanical survey. This was conducted during the rainy season. The study area was divided into 1 km grids on a map. From among these, 10 grids were randomly selected for the study. A Global Positioning System (GPS) receiver was used to locate the grids in the study area. In each grid, three parallel transect lines separated by 100 m, each measuring 1000 m running south to north were established. Along each line 10 plots spaced 50 m apart on alternating sides of the line were established. A total of 286 plots each measuring 40 × 20 m were established. To avoid the edge effect, the first plot was established 5 m away from a trail. The main plot was subdivided into sub-plots to ease sampling effort. All woody tree species encountered in the plot were identified, recorded and their diameter at breast height (dbh) or root collar diameter (for young or very small individuals) measured (dbh was used in coverage computations described below). Individuals with sizes greater than 20 cm dbh, were measured over the entire plot (40 × 20 m); those between 10 – 19 cm dbh in the 20 × 20 m sub-plot; and those between 5 – 9 cm, in the 20 × 10 subplot. Smaller plants, 1 – 4 cm, were measured in the 10 × 10 m subplot. The minimum diameter measured was 1 cm. We computed relative densities (RD) of all trees encountered, as well as the relative frequencies (RF) and relative coverage (RC). These indices were summed up into a composite importance value index (IVI), as described by Muthuramkumar and Parthasarathy (2000). The RD describes the abundance of a species, while the RF describes the extent of distribution of a species, and the RC describes the area occupied by a species which is influenced by the size of the plant. We computed density of species as the number of individuals of a species occupying a unit area, frequency as the proportion of plots occupied by a species and coverage as the area occupied by a species for the sampled area. From these indices proportions or relative indices (RD, RF and RC) were computed and summed up to generate the composite importance value index (IVI) which demonstrates the relative ecological importance of a species.

## Results

### Socio-demographic characteristics of the goat farmers

The mean age of the respondents was 37 years (range 12 - 68 years). Almost all farmers interviewed were married and had attained primary education (Table 1). They stated that crop and livestock farming were their major occupations and therefore their main source of income.

**Table 1:** **Socio-demographic characteristics of Buyende District, Uganda (n = 139)**

**Characteristic**	**Percentage**	**Characteristic**	**Percentage**
Marital status		Main occupation	
Married	80	Crop and livestock farming	83
Single	13	Small scale trade	9
Widowed	6	Civil servants	3
Divorced	1	Others*	5
Education			
None	12		
Primary	51		
O-level	32		
A-level	1		
Tertiary	4		

*student, fishing, employed in private company.

### Goat rearing practices by households

**Figure 1:**
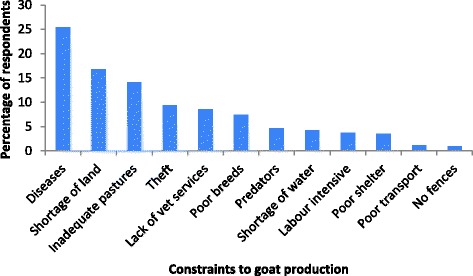
Challenges faced in goat production by farmers in Buyende District, Uganda.

The mean flock size of goats reared by farmer households was 13 goats. Indigenous breeds were most common (72%) followed by crosses between indigenous breed and the South African Boer goats (25%) and a few exotic breeds (3%). The main reason (87%) for rearing goats was that they were a form of insurance against hard times. Ownership (65%) of goats, selling (76%) and decision making (55%) on proceeds from the goats was the responsibility of men. But the feeding of goats (66%) was the responsibility of women. Tethering was the major goat feeding practice (90%) in the area followed by free range (9%) and cut and carry system of feeding (1%). Goats were kept in the house at night by most respondents (55%). The key challenges facing goat production were diseases, shortage of land and inadequate pastures (Figure 1).

### Goat feed resources

Farmers stated that they fed their goats with different feed resources with the main feed being natural pastures (70%). Other feed resources were crop residues (15%: sweet potato vines, maize, millet or sorghum stover, and rice straw), and food crop by-products (12%: sweet potato peels, banana peels and cassava peels). A few farmers used compound feed (3%). Banana crop peelings were not much utilized as goat feed because bananas are scarce in the area of study. On the other hand, sweet potato peels were abundant because sweet potato is the common food crop in the area. However, these were not commonly used because goats are known to die when they eat large quantities of potato peelings and then water. For this reason only small quantities of sweet potato peelings were given to the goats. Most farmers reported that they do not supplement (58%), conserve (84%) or treat (84%) goat feeds. These were mainly attributed to lack of knowledge (52%) and lack of money (37%). The few who supplemented goat feed, only sprinkled common salt to food crop peelings (63%).

Concerning the pasture species, farmers have observed that goats browse and graze on 48 species distributed in 18 families and 39 genera (Table 2). The growth habit of the pasture species were mostly trees (24) and shrubs (11). Herbaceous species i.e., herbs and grasses contributed 4 and 9 species, respectively. The most frequently mentioned browse species were *Ficus natalensis*, *Harrisonia abyssinica*, *Acalypha psilostachya*, *Artocarpus heterophyllus* and *Lantana camara* while *Panicum maximum* and *Impeata cylindrica* were the most mentioned herbacious species (Table 2). Some of these species were known to be exploited for other products. For example *Ficus natalensis* is used as fire wood, human medicine and as raw material for making backcloth*. Mangifera indica* is used as food for humans, firewood and timber.

**Table 2:** **Pasture species reported by respondents of Buyende district (Uganda) as being utilized by goats. a. woody species and b. herbaceous species**

**Species (Voucher no.)**	**Local name**	**Family**	**Growth form**	**No of respondents**
a. Woody species				
*Ficus natalensis* Hochst. (NDK 59)	Mugaile	Moraceae	Tree	101
*Harrisonia abyssinica* Oliv. (NDK 35)	Ndalike/ensaikye	Rutaceae	Shrub	74
*Acalypha psilostachya* Hochst. ex A.Rich. (NDK 36)	Empelele	Euphorbiaceae	Shrub	43
*Artocarpus heterophyllus* Lam. (NDK 20)	Fene	Moraceae	Tree	35
*Lantana camara* L*.* (NDK 60)	Kapanga	Verbenaceae	Shrub	25
*Mangifera indica* L. (NDK 34)	Muyembe	Anacardiaceae	Tree	20
*Acacia senegal* (L.) Willd. (NDK 11, NDK 41)	Katasubwa/Budali	Fabaceae	Tree	19
*Citrus sinensis* (L.) Osbeck (NDK 19)	Muchungwa	Rutaceae	Tree	17
*Acacia sieberiana* DC. (NDK 48)	Melamaino	Fabaceae	Tree	16
*Albizia zygia* (DC.) J.F. Macbr*.* (NDK 24)	Mulongo	Fabaceae	Tree	15
*Rhus natalensis* Krauss (NDK 8)	Musese/kasakasaka	Anacardiaceae	Shrub	12
*Flueggea virosa* (Roxb. ex Willd.) Royle (NDK 30)	Nkandwa	Phyllanthaceae	Shrub	11
*Milicia excelsa* (Welw.) C.C. Berg (NDK 50)	Muvule	Moraceae	Tree	11
*Faidherbia albida* Del*.* (NDK 9)	Bwatampasa	Fabaceae	Tree	9
*Lannea schweinfurthii* (Engl.) Engl. (NDK 23)	Musinga bakali	Anacardiaceae	Tree	6
*Capparis fascicularis* DeWolf (NDK 7)	Namukodolya	Capparaceae	Shrub	6
*Acacia polycantha* Willd*.* (NDK 45)	Mukongoito	Fabaceae	Tree	5
*Melia azedarach* L*.* (NDK 42)	Kabalila	Meliaceae	Tree	4
*Urena lobata* L. subsp.*sinuata* (L.) Borss. Waalk(NDK 52)	Biwanda	Malvaceae	Shrub	4
*Acacia brevispica* Harms (NDK 22)	Empule	Mimoseae	Tree	3
*Vernonia amygdalina* Delile (NDK 21)	Mubilili	Asteraceae	Shrub	3
*Maesopsis eminii* Engl. (NDK 14)	Musizi	Rhamnaceae	Tree	4
*Acacia Seyal* Delile (NDK 56)	Mufuwanduzi	Fabaceae	Tree	3
*Tamarindus indica* L. (NDK 15)	Mukooge	Fabaceae	Tree	2
*Combretum molle* R. Br. ex G. Don, (NDK 65)	Mukoola	Combretaceae	Tree	2
*Grewia similis* K. Schum. (NDK 25)	Sinya	Malvaceae	Shrub	2
*Tinnea aethiopica* Kotschy ex Hook. f. (NDK 3)	Muzaimwa	Lamiaceae	Shrub	2
*Azadirachta indica* A. Juss. (NDK 5)	Neem	Meliaceae	Tree	3
*Senna siamea* (Lam.) H.S. Irwin & Barneby (NDK 12)	Gasia	Fabaceae	Tree	1
*Euphorbia tirucalli* L. ( NDK 49)	Lukone	Euphorbiaceae	Shrub	1
*Psidium guajava* L. (NDK 18)	Mapera	Myrtaceae	Tree	1
*Albizia coriaria* Welw. ex Oliv. (NDK 43)	Musita	Fabaceae	Tree	1
*Ficus sycomorus* L. (NDK 31)	Mukunyu	Moraceae	Tree	1
*Eucalyptus grandis* Maiden (NDK 40)	Kalitunsi	Myrtaceae	Tree	1
*Ficus thonningii* Blume (NDK 57)	Kiryanonyi	Moraceae	Tree	1
b. herbaceous species				
Species (Voucher no.)	Local name	Family	Growth form	No of respondents
*Panicum maximum* Döll (NDK 63)	Mukonzi	Poaceae	Grass	87
*Impeata cylindrica* (L.) Beauv. C. E. Hubbard (NDK 68)	Ibembe	Poaceae	Grass	52
*Sporobolus pyramidalis* P. Beauv. (NDK 66)	Kasibante	Poaceae	Grass	26
*Conyza floribunda* Kunth (NDK 64)	Katikati	Asteraceae	Herb	23
*Cynodon dactylon* (L.) Pers. (NDK 62)	Lufafa	Poaceae	Grass	15
*Solanum incanum* L*.* (NDK 54)	Entulatula	Solanaceae	Herb	14
*Commelina benghalensis* L. (NDK 67)	Nanda	Commelinaceae	Herb	12
*Euphorbia heterophylla* L. (NDK 61)	Kafadanga	Euphorbiaceae	Herb	6
*Rottboelia cochinchinensis* (Lour. ) W. D. Clayton (NDK 58)		Poaceae	Grass	3
*Sporobolus africanus* (Poir.) Robyns & Tourney (NDK 47)		Poaceae	Grass	2
*Echinochloa colona* (L.) Link (NDK 46)		Poaceae	Grass	1
*Digitaria abyssinica* Stapf (NDK 33)	Lumbugu	Poaceae	Grass	1
*Chloris gayana* Kunth (NDK 10)	Mulifinyara	Poaceae	Grass	1

**Figure 2:**
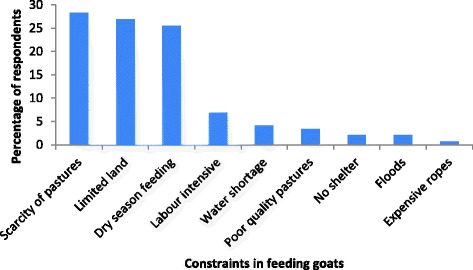
Challenges faced in feeding goats by farmers in Buyende District, Uganda.

The key goat feeding constraints according to farmers were scarcity of pastures, especially in the dry season, and small plots of land (Figure 2). When asked how the challenge can be overcome, respondents suggested introduction of drought resistant pastures and receiving training in skills of feed conservation, treatment and supplementation especially during the dry season.

### Farmers’ perception about the seasonal availability of feed resources

According to farmers, herbaceous species availability is restricted to the wet season (Table 3). However, during the wet season, farmers plant their crops. For this reason, the goats are tethered to stop them from straying into crop fields. This therefore limits the goats’ freedom to graze optimally. In the dry season, grasses and herbs dry up, to leave only crop residues, food crop by-products and browses as the main feed resource for the goats. Feed availability becomes restricted further towards the end of the dry season because at this time even food peelings are not available. Furthermore, at this time crop residues are cleared and burnt to prepare gardens for the next rain season. At this point browses are the major feed resources available to the goats. The goats are left to freely graze since there are no crops in the gardens.

**Table 3:** **Feeding calendar and seasonal availability of feed resources in Buyende District, Uganda**

**Months**	**J**	**F**	**M**	**A**	**M**	**J**	**J**	**A**	**S**	**O**	**N**	**D**
**Season**	***Dry***	***Dry***	***Dry***	***Wet***	***Wet***	***Wet***	***Dry***	***Dry***	***Wet***	***Wet***	***Dry***	***Dry***
Agriculture activity	*BC*	*BC*	*BC*	*BC*	*P*	*W*	*HA*	*HA*	*P*	*P*	*HA*	*HA*
	*BU*	*BU*	*BU*	*P*	*W*		*BC*	*BC*	*W*	*W*		
	*HA*											
Availability of feed resources												
Trees and shrubs	*	*	*	*	*	*	*	*	*	*	*	*
fresh grass				*	*	*			*	*		
dry grass	*	*										*
crop residues	*	*					*	*			*	*
food peelings	*						*	*	*	*	*	*

BC – bush clearing, BU- burning, HA- harvesting, P – planting, W- weeding, *- Feed resource availability, *preferred feed resource.

### Abundance of the browse species

We conducted an ecological survey (restricted to browse species) to determine the availability of species mentioned in the ethnobotanical survey. From the ecological survey we encountered 31 out of the 35 browse species mentioned in the ethnobotanical survey. The species that we did not encounter were *Acalypha psilostachya*, *Acacia abyssinica*, *Psidium guajava* and *Eucalyptus grandis.* We computed an Importance Value Index (IVI) to determine the most available species. The IVI value ranged between 0.3 and 60. The species with the highest IVI included *Combretum molle*, *Lantana camara*, *Albizia zygia*, *Mangifera indica*, and *Albizia coriaria*. Their high IVI values were contributed by their being abundant and widely distributed (Table 4). Some of the species known to be highly preferred by goats were rare according to the computed IVI (i.e. less than 30%).

**Table 4:** **The browse species found at Buyende District (Uganda) in decreasing order of importance value index (IVI = RC + RD + RF)**

**Species**	**RC (%)**	**RD (%)**	**RF (%)**	**IVI**
*Combretum molle*	5.7	15.1	39.2	60.0
*Lantana camara*	0.5	16.0	39.2	55.7
*Albizia zygia*	5.2	5.7	26.9	37.8
*Mangifera indica*	8.4	0.9	22	31.3
*Albizia coriaria*	6.9	1.5	21.7	30.1
*Milicia excelsa*	9.2	0.7	19.9	29.8
*Rhus natalensis*	2.0	3.5	23.1	28.6
*Harrisonia abyssinica*	0.4	5.9	19.9	26.2
*Ficus sycomorus*	10.0	0.2	12.2	22.4
*Ficus natalensis*	8.4	0.4	12.2	21.0
*Acacia sieberiana*	0.7	2.0	16.4	19.1
*Flueggea virosa*	0.2	2.8	15.7	18.7
*Lannea schweinfurthii*	6.0	0.5	9.4	15.9
*Artocarpus heterophyllus*	2.6	0.4	8.0	11.0
*Acacia polycantha*	1.4	1.2	7.0	9.6
*Tinnea aethiopica*	0.1	2.6	5.9	8.6
*Tamarindus indica*	1.4	0.2	5.9	7.5
*Euphorbia tirucalli*	0.8	0.4	6.3	7.5
*Acacia senegal*	0.5	1.1	5.2	6.8
*Acacia albida*	0.0	1.4	3.5	4.9
*Vernonia amygdalina*	0.5	0.4	3.8	4.7
*Ficus thonningii*	2.0	0.1	2.4	4.5
*Melia azedarach*	0.5	0.2	2.8	3.5
*Maesopsis eminii*	0.5	0.1	2.4	3.0
*Citrus sinensis*	0.1	0.1	2.4	2.6
*Grewia similis*	0.0	1.5	0.7	2.2
*Acacia brevispica*	0.0	0.2	1.0	1.2
*Azadirachta indica*	0.0	0.0	0.7	0.7
*Senna siamea*	0.1	0.2	0.3	0.6
*Capparis fascicularis*	0.0	0.0	0.3	0.3
*Senegalia senegal*	0.0	0.0	0.3	0.3

RC = relative coverage, RD = relative density, RF = relative frequency; IVI = Importance Value Index.

## Discussion

The goat rearing practices in Buyende district are characterized by tethering in natural pastures. This is a common practice that is practiced to stop goats from straying into crop gardens to destroy crops, in places where land is limiting and there is mixed crop and livestock production systems (Kosgey 2004). In this study, diseases were the major challenge affecting goat production. The major diseases in this area are vector bone diseases like trypanosomiasis, internal and external parasites and foot and mouth disease (Waiswa *et al*. 2006; Kasambula *et al.*^2012^). Shortage of land and inadequate pastures in terms of quality and quantity were also key challenges facing goat production. This is not unique to Uganda and is not surprising given the growing human population. Increasingly less land is left for grazing which in turn limits the quantity of pastures available for the goats. This therefore calls for introducing or improving feeding technologies that can be used amidst the prevailing challenges.

The nature in which the goats are tethered in the study area limits the quality and quantity of feed that they actually eat and this in turn reduces their productivity. This is because the goats are fastened on one spot for the entire day and have access to only the feed that is available in the radius of the rope. Studies conducted by Muir *et al*. (1995) in Mozambique indicated that supplementation of tethered animals with leguminous tree foliage improved the average daily weight gains. There is therefore a need to improve the quality of nutrition for goats that are tethered in natural pastures using supplementation from locally available leguminous tree foliage in order to improve their productivity.

Feed resource availability is seasonal; in the wet season goats have abundant feed in the form of herbaceous species and browses. But then goats are not allowed to graze freely in the wet season for fear of destroying crops. This limits the amount of feed available to the goats especially in the tethering system of feeding. In the dry season when goats are allowed to graze freely, feed availability is low since most forage has dried out and all that is available are crop residues. In the dry season, the only green forage is the browse from trees and shrubs (Tolera and Said 1997).

Although crop farming interferes with goat rearing, in the dry season, crop residues and food crop by-products are fed to goats in addition to the browses which are the main feed resource. The key challenge with the crop residues is that they are high in fibre and low in crude protein and therefore provide low nutrition value to the goats (Katongole *et al*. 2008). Additionally, the commonly available food crop by-products of sweet potato and cassava peels are not recommended to be fed in high amounts because they are toxic and can easily cause death of the goats if their intake is not regulated. The deaths could be due to the presence of oxalate and trypsin inhibitors in sweet potatoes (Bradbury *et al.*^1992^, Ravindran *et al.*^1995^) and cyanogenic glucosides in cassava peels (Tweyongyere and Katongole 2002). Browse species on the other hand have a higher crude protein content, lower fibre and are more digestible compared to the grasses and crop residues (Evitayani *et al*. 2004). Browse species therefore provide a better feed resource during the dry season in areas where they are available.

Natural pastures, especially trees and shrubs, are the main goat feed resource. The species browsed on are well known goat feed and have been reported in East Africa and elsewhere. Species already reported as fodder species in Uganda include *Ficus natalensis* (Kabasa *et al*. 2004), *Harrisonia abyssinica* (Mukadasi *et al*. 2008), *Acalypha psilostachya*, *Artocarpus heterophyllus, Conyza floribunda, Albizia zygia* and *Mangifera indica* (Tabuti and Lye 2009). *Lantana camara* has been reported in Kenya (Roothaert and Franzel 2001), *Citrus sinensis* in Nigeria (Okoli *et al*. 2003), *Acacia sieberiana* in South Africa (Mokoboki *et al*. 2011), *Acacia senegal* in Burkina Faso (Sanon *et al*. 2008) and *Rhus natalensis* in Ethiopia (Shenkute *et al*. 2012). Species that are widely used, by humans or animals, indicate their value as safe and useful species. The species that are known as browse/fodder species from a wide geographical area should form a short list from which feeds should be developed and promoted for feeding goats. Out of the 31 browse species encountered during the ecological survey, some species mentioned to be the most preferred for goats by the community were rare according to the computed IVI i.e. less than 30%. This suggests that interventions to propagate the rare species are called for.

## Conclusion

In conclusion, the rearing practices of goats in Buyende district are comprised of indigenous goats tethered in natural pastures especially browses which stay longer through the dry season. However, some of the most preferred browses are rare according to the computed IVI (i.e. less than 30%).

There is a need to conduct nutrition evaluation studies on the preferred browse species identified in this study before they can be used in the development of rations to improve the quality of goat nutrition. There is a need to also determine the germination characteristics and seedling establishment characteristics of the preferred browse species with an aim of domesticating them, to ensure a sustainable supply of quality feeds to the goats throughout the year and ultimately improve goat productivity.
